# Using Calibration to Reduce Measurement Error in Prevalence Estimates Based on Electronic Health Records

**DOI:** 10.5888/pcd15.180371

**Published:** 2018-12-13

**Authors:** Pui Ying Chan, Yihong Zhao, Sungwoo Lim, Sharon E. Perlman, Katharine H. McVeigh

**Affiliations:** 1 Division of Epidemiology, New York City Department of Health and Mental Hygiene, Long Island City, New York; 2 Department of Health Policy and Health Services Research, Henry M. Goldman School of Dental Medicine, Boston University, Boston, Massachusetts; 3 Division of Family and Child Health, New York City Department of Health and Mental Hygiene, Long Island City, New York

## Abstract

**Introduction:**

Increasing adoption of electronic health record (EHR) systems by health care providers presents an opportunity for EHR-based population health surveillance. EHR data, however, may be subject to measurement error because of factors such as data entry errors and lack of documentation by physicians. We investigated the use of a calibration model to reduce bias of prevalence estimates from the New York City (NYC) Macroscope, an EHR-based surveillance system.

**Methods:**

We calibrated 6 health indicators to the 2013–2014 NYC Health and Nutrition Examination Survey (NYC HANES) data: hypertension, diabetes, smoking, obesity, influenza vaccination, and depression. We classified indicators into having low measurement error or high measurement error on the basis of whether the proportion of misclassification (ie, false-negative or false-positive cases) was greater than 15% in 190 reviewed charts. We compared bias (ie, absolute difference between NYC Macroscope estimates and NYC HANES estimates) before and after calibration.

**Results:**

The health indicators with low measurement error had the same bias after calibration as before calibration (diabetes, 2.5 percentage points; smoking, 2.5 percentage points; obesity, 3.5 percentage points; hypertension, 1.1 percentage points). For indicators with high measurement error, bias decreased from 10.8 to 2.5 percentage points for depression, and from 26.7 to 8.4 percentage points for influenza vaccination.

**Conclusion:**

The calibration model has the potential to reduce bias of prevalence estimates from EHR-based surveillance systems for indicators with high measurement errors. Further research is warranted to assess the utility of the current calibration model for other EHR data and additional indicators.

## Introduction

Electronic health record (EHR) systems have been increasingly adopted in the United States (1). In addition to being a useful tool for health care providers, EHRs contain rich clinical data, which allow for possible public health applications, such as monitoring diseases. The distinct advantage of EHRs in potentially providing near real-time and area-specific data at a relatively low cost is appealing to public health practitioners (2,3) and has encouraged the development of EHR-based surveillance (4–8). However, EHR-based prevalence estimates may be subject to selection and misclassification biases. EHR data are derived from convenience samples of medical practices. They also may underrepresent uninsured and healthy people, who may not visit physicians regularly (9–11). Selection bias may be addressed by poststratification, which applies weight factors to adjust for bias introduced by sampling imbalances in the target population. Misclassification bias, or measurement error, may arise as a result of data entry errors, inconsistent screening practices or documentation of health conditions among physicians, no documentation for out-of-facility services, and inability to capture information in unstructured fields (9,12–14). Researchers have found high sensitivity and specificity for some conditions (eg, diabetes) (4,7) but not others (eg, depression) (8,15) in EHRs. To our knowledge, however, few researchers have explored methods for adjusting estimates when high measurement error exists.

In this study, we examined the use of a calibration model to correct for measurement error in the prevalence estimates from the New York City Macroscope (NYC Macroscope), an EHR-based surveillance system. Calibration is a well-established approach to correcting measurement error in self-reported survey data (16,17). Briefly, a calibration model predicts the true disease status on the basis of data that have gold standard measurements, and the resulting model is used to adjust for the biased status. We hypothesized that such a calibration model could reduce bias of prevalence estimates for indicators with high measurement error in the NYC Macroscope.

## Methods

### Data sources

The NYC Macroscope is an EHR-based surveillance system for chronic disease and risk factors developed by the New York City Department of Health and Mental Hygiene (NYC DOHMH) in 2012 (18). It uses aggregate count data (eg, the number of patients with hypertension) from the EHRs of ambulatory primary care providers in New York City who have agreed to share data with the NYC DOHMH and who meet criteria for documentation quality aligned with the US government’s stage 1 meaningful use incentive program (19) (eg, ICD-9 [*International Classification of Diseases, Ninth Revision*] diagnoses recorded for at least 80% of patients seen). The NYC Macroscope has limited data on stratifying variables or covariates (eg, age, sex) as a result of technical limitations of the query system. The aggregate count data are converted into person-level data, where each row represents a (de-identified) patient record, during data processing. In this study, we used data from the 2013 NYC Macroscope (7,8), which included 392 practices and 716,076 patients aged 20 or older who visited their provider at least once in 2013.

The 2013–2014 New York City Health and Nutrition Examination Survey (NYC HANES) provided the gold standard data for this study. The 2013–2014 NYC HANES was an in-person examination survey that consisted of survey questions and objectively measured health data (through physical examination and laboratory testing) for 1,527 noninstitutionalized New York City residents aged 20 or older (20). Of these participants, 1,135 reported visiting a health care provider in the previous year (ie, were in care). For a subset (n = 190) of these participants, EHRs were abstracted from the primary care provider for the period of January 1, 2011, through the 2013–2014 NYC HANES interview date (August 2013 through June 2014) (21). The algorithms for defining NYC Macroscope indicators were applied to the abstracted data, allowing assessment of measurement error in NYC Macroscope indicators against the 2013–2014 NYC HANES gold standard measures without linking the 2 data sources. Details of the chart review study are available elsewhere (21). The 2013–2014 NYC HANES was approved by the NYC DOHMH and City University of New York School of Public Health institutional review boards, and the chart review study was approved by the NYC DOHMH institutional review board.

### Measures

#### Indicators

Seven health indicators were available in the 2013 NYC Macroscope, including obesity, smoking, diabetes, hypertension, hyperlipidemia, influenza vaccination, and depression. We included all indicators except hyperlipidemia in our analysis. The previous validation study of 2013 NYC Macroscope against 2013–2014 NYC HANES revealed poor performance on both sensitivity and specificity for hyperlipidemia (21). After considering the possibility of undercount of hyperlipidemia cases in the 2013–2014 NYC HANES (21), we decided not to include hyperlipidemia in our study. Furthermore, 2 definitions for hypertension and diabetes exist in the NYC Macroscope: “diagnosis” (based on diagnosis only) and “augmented” (based on diagnosis, medication, and objective measures [ie, blood pressure and laboratory tests]). In this analysis, we used the more inclusive augmented definition. The indicator definitions for 2013 NYC Macroscope were developed in consideration of both sufficiently capturing data available in the EHRs and adequate alignment with the 2013–2014 NYC HANES data for validation purposes (18). The indicator definitions for 2013 NYC Macroscope and 2013–2014 NYC HANES can be found in previous NYC Macroscope validation studies (7,8,21) and are the following:

**Hypertension.** NYC Macroscope: an ICD-9 code for hypertension ever recorded in the EHR, the last systolic blood pressure of 140 mm Hg or greater or a diastolic blood pressure of 90 mm Hg or greater in 2013, or a prescription for an antihypertension medication in 2013. NYC HANES: a measured systolic blood pressure of 140 mm Hg or greater or a measured diastolic blood pressure of 90 mm Hg or greater or reported to ever have been diagnosed with hypertension by a health care professional.

**Diabetes.** NYC Macroscope: an ICD-9 code for diabetes ever recorded in the EHR, the last glycated hemoglobin (HbA_1c_) measurement of 6.5 or greater in 2012–2013, or a prescription for a diabetes medication in 2013. NYC HANES: a measured HbA_1c_ of 6.5 or greater or reported to ever have been diagnosed with diabetes by a health care professional.

**Obesity.** NYC Macroscope: the last recorded body mass index of 30.0 or more in the EHR in 2013. NYC HANES: Body mass index is 30.0 or more, calculated by dividing measured weight in kilograms divided by measured height in meters squared.

**Smoking.** NYC Macroscope: an indication of current smoking in the last recorded structured field for smoking status in 2013. NYC HANES: reported to have smoked 100 or more cigarettes in lifetime and be currently smoking every day or some days.

**Influenza vaccination.** NYC Macroscope: a relevant seasonal influenza vaccination ICD-9 code, CPT (Current Procedural Terminology) code, or CVX (vaccine administered) code recorded in the EHR in 2013. NYC HANES: reported to have received seasonal influenza vaccination in the previous year.

**Depression.** NYC Macroscope: an ICD-9 code for depression ever recorded in the EHR or a score of 10 or more on the Patient Health Questionnaire (PHQ-9) in 2013. NYC HANES: a PHQ-9 score of 10 or more at the interview or reported to ever have been diagnosed with depression by a health care professional.

The lookback period of the chart review study differed slightly from the lookback period of the 2013 NYC Macroscope (21). For obesity, smoking, influenza vaccination, blood pressure (hypertension), medication (hypertension and diabetes), and PHQ-9 (depression), the lookback period was 1 year before the participant’s NYC HANES interview date. For HbA_1c_ (diabetes), the lookback was 2 years before the NYC HANES interview date.

#### Dependent and independent variables

The dependent variable in our regression model was indicator status in the 2013–2014 NYC HANES. The independent variables were indicator status in the chart review data and all covariates available in the 2013 NYC Macroscope data (7,8), which included age group (20–39 y, 40–59 y, or 60–100 y), sex (male or female), and neighborhood poverty, defined as the proportion of households in one’s residential ZIP code with an annual income below the US federal poverty threshold per the 2008–2012 American Community Survey (<10%, 10% to <30%, or ≥30%) (22).

### Quantifying measurement error

We treated indicator status in the 2013–2014 NYC HANES as the gold standard measure. For each health indicator, the EHR measures (from the chart review data) that deviated from the 2013–2014 NYC HANES measures were coded as misclassified (ie, false-positive or false-negative cases). We considered an indicator as having high measurement error when the proportion of misclassification was greater than 15%.

### Statistical analysis

For each indicator, we calibrated the NYC Macroscope prevalence estimate by using the following steps:

**Step 1.** In the chart review sample (n = 190), we conducted Firth logistic regression (23) to predict a positive status (eg, having hypertension) in the 2013–2014 NYC HANES, and we used the independent variables and their possible interactions as predictors. We used stepwise selection to choose an optimal set of predictors. We required a significance level of .40 for a variable to be included in the model and a significance level of 0.50 for a variable to be retained in the model. Our primary goal in this step was to find a model with a high prediction accuracy, not to find predictors that were significantly associated with the outcome. After obtaining predicted probabilities from the model, we chose an optimal probability cutoff for classifying a person’s indicator status such that the Youden *J* index (24) (sensitivity + specificity −1) was maximized (Table 1). We used this probability cutoff to reclassify a patient’s indicator status in the NYC Macroscope in a later step. 

**Table 1:** Specification of Logistic Regression Models in the Chart Review Sample for Predicting a Positive Indicator Status From the 2013–2014 NYC HANES and the Selected Probability Cutoff

Indicator	Independent Variables Used	Selected Probability Cutoff^d^
Obesity	Indicator status on chart, sex, age group (20–39 y, 40–59 y, 60–100 y), indicator status on chart × sex	.677
Smoking	Indicator status on chart	.891
Diabetes^a^	Indicator status on chart, age group (20–39 y, 40–59 y, 60–100 y)	.808
Hypertension^b^	Indicator status on chart, sex, age group (20–39 y, 40–59 y, 60–100 y), neighborhood poverty^c^ (<10%, 10% to <30%, ≥30%), indicator status on chart × sex, sex × age group	.470
Influenza vaccination	Indicator status on chart, sex, neighborhood poverty^c^ (<10%, 10% to <30%, ≥30%)	.550
Depression	Indicator status on chart, sex, age group (20–39 y, 40–59 y, 60–100 y), neighborhood poverty^c^ (<10%, 10% to <30%, ≥30%)	.281

Abbreviation: NYC HANES, New York City Health and Nutrition Examination Survey.

a Augmented definition. For the chart review sample, definition was based on diagnosis, glycated hemoglobin (HbA_1c_), and medication; for 2013–2014 NYC HANES, it was based on diagnosis and measured HbA_1c_.

b Augmented definition. For the chart review sample, definition was based on diagnosis, blood pressure, and medication; for 2013–2014 NYC HANES, it was based on diagnosis and measured blood pressure.

c Proportion of households in a ZIP code area living below the US federal poverty threshold per the 2008–2012 American Community Survey.

d Based on the highest value for the Youden index (sensitivity + specificity −1).

**Step 2.** We plugged in the model coefficients (obtained in Step 1) to the NYC Macroscope sample (n = 716,076) to calculate the predicted probability of having a positive indicator status for each individual:

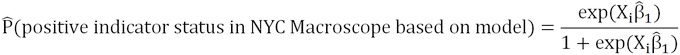



where Χ_i_ and 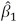 are vectors of selected covariates and model coefficients, respectively.

**Step 3.** We reclassified each person’s indicator status on the basis of whether or not the calculated probability exceeded the corresponding cutoff (obtained in Step 1), and we obtained a calibrated estimate based on the new classification.

After calibration, we assessed change in bias, defined as the absolute difference between the NYC Macroscope estimate and 2013–2014 NYC HANES estimate, for each indicator. We obtained the 2013–2014 NYC HANES estimates from the in-care participants (n = 1,135) and poststratified NYC Macroscope estimates to the 2013–2014 NYC HANES in-care population. All estimates were age-adjusted to the US 2000 standard population. The 2013–2014 NYC HANES and noncalibrated NYC Macroscope estimates and the sample characteristics of the 2013 NYC Macroscope were previously published (7,8,21). Traditionally, a data set independent of the data set used for model building would be used for validation (ie, in an assessment of bias), but in this study, the 2 data sets overlapped (ie, the 190 chart review participants were a subset [17%] of the 2013–2014 NYC HANES in-care sample). As a sensitivity analysis, we calculated 2013–2014 NYC HANES estimates from the in-care participants who were not in the chart review study (n = 945) and examined changes in the estimates. All analyses were performed by using SAS version 9.4 (SAS Institute Inc) and SUDAAN version 11.0 (RTI International).

## Results

The NYC Macroscope sample was similar to the chart review sample in age distribution: about one-fourth were adults aged 60 or older. Women were the majority in both samples, but the proportion of women was lower in the NYC Macroscope sample (59% vs 65%). A smaller proportion in the NYC Macroscope sample (14%) than in the chart review sample (24%) lived in the wealthiest neighborhoods (neighborhood poverty <10%).

The proportion of misclassification varied across indicators (Table 2): 6% for obesity; 4% for smoking; 3% for diabetes; 13% for hypertension; 31% for influenza vaccination; and 19% for depression. Influenza vaccination and depression were categorized as indicators with high measurement error, and the others were categorized as indicators with low measurement error.

**Table 2:** Number of True-Positive, True-Negative, False-Positive, and False-Negative Cases for Each Health Indicator in a Comparison of Chart Review Data (N = 190) and 2013–2014 NYC HANES Data

Indicator	Effective Sample Size^a^	True Positive	True Negative	False Positive	False Negative	Proportion of Misclassification^d^, %	Measurement Error^e^
Obesity	159	51	98	5	5	6	Low
Smoking	151	20	125	2	4	4	Low
Diabetes^b^	179	32	142	3	2	3	Low
Hypertension^c^	190	64	102	9	15	13	Low
Influenza vaccination	189	52	78	5	54	31	High
Depression	189	16	138	3	32	19	High

Abbreviation: NYC HANES, New York City Health and Nutrition Examination Survey.

a Effective sample size for some indicators was not 190 because of missing data in either 2013–2014 NYC HANES or chart review.

b Augmented definition. For the chart review sample, definition was based on diagnosis, glycated hemoglobin (HbA_1c_), and medication; for 2013–2014 NYC HANES, it was based on diagnosis and measured HbA_1c_.

c Augmented definition. For the chart review sample, definition was based on diagnosis, blood pressure, and medication; for 2013–2014 NYC HANES, it was based on diagnosis and measured blood pressure.

d Proportion of the sum of the number of false-positive cases and false-negative cases.

e Categorized as high when the proportion of misclassification was greater than 15% and low otherwise.

For the indicators with low measurement error, the NYC Macroscope prevalence estimates did not change after calibration for diabetes (15.3%; bias, 2.5 percentage points), obesity (27.8%; bias, 3.5 percentage points), and smoking (15.2%; bias, 2.5 percentage points) (Table 3). The NYC Macroscope prevalence estimate for hypertension increased from 39.2% before calibration to 41.4% after calibration, but its bias did not change (1.1 percentage points). The influenza vaccination prevalence estimate increased from 20.9% before calibration to 39.2% after calibration, and bias decreased from 26.7 percentage points to 8.4 percentage points. The depression prevalence estimate increased from 8.2% before calibration to 21.5% after calibration, and bias decreased from 10.8 percentage points to 2.5 percentage points. Our sensitivity analysis showed a small degree of change in the 2013–2014 NYC HANES estimates when the chart review participants were excluded; these changes ranged from a decrease of 1.2 percentage points (from 47.6% to 46.4%) for influenza vaccination to an increase of 0.6 percentage points (from 17.7% to 18.3%) for smoking.

**Table 3:** Prevalence Estimates From 2013–2014 NYC HANES^a^ and 2013 NYC Macroscope^b^ (Before Calibration and After Calibration) and Bias^c^ of the NYC Macroscope Estimates

Indicator	2013–2014 NYC HANES	% (Bias^c^)	
		2013 NYC Macroscope, Before Calibration^d^	2013 NYC Macroscope, After Calibration
Obesity	31.3	27.8 (3.5)	27.8 (3.5)
Smoking	17.7	15.2 (2.5)	15.2 (2.5)
Diabetes^e^	17.8	15.3 (2.5)	15.3 (2.5)
Hypertension^f^	40.3	39.2 (1.1)	41.4 (1.1)
Influenza vaccination	47.6	20.9 (26.7)	39.2 (8.4)
Depression	19.0	8.2 (10.8)	21.5 (2.5)

Abbreviation: NYC HANES, New York City Health and Nutrition Examination Survey.

a Based on previously published data for 1,135 NYC HANES participants who reported seeing a health care provider in the past year. Data sources: Thorpe et al (7) and McVeigh et al (8).

b The NYC Macroscope is an electronic health record-based surveillance system for chronic disease and risk factors developed by the NYC Department of Health and Mental Hygiene (18).

c Quantified as the absolute difference between the NYC Macroscope and 2013–2014 NYC HANES prevalence estimates in percentage points.

d Data sources: Thorpe et al (7) and McVeigh et al (8).

e Augmented definition. For NYC Macroscope, definition was based on diagnosis, glycated hemoglobin (HbA_1c_), and medication; for 2013–2014 NYC HANES, it was based on diagnosis and measured HbA_1c_.

f Augmented definition. For NYC Macroscope, it was based on diagnosis, blood pressure, and medication; for 2013–2014 NYC HANES, it was based on diagnosis and measured blood pressure.

## Discussion

In this study, we calibrated prevalence estimates from the NYC Macroscope for 6 health indicators by using data from a well-established survey, the 2013–2014 NYC HANES, as the reference data. As expected, calibration had no effect or limited effect on the bias of prevalence estimates for indicators with low measurement error, but calibration reduced bias in prevalence estimates for indicators with high measurement error. The improvement was substantial for depression prevalence estimates, for which we found that bias was reduced to 2.5 percentage points from 10.8 percentage points. Bias was also reduced for influenza vaccination prevalence estimates, by 18.3 percentage points, from 26.7 percentage points to 8.4 percentage points.

Our results were consistent with our expectations that calibration could reduce bias in prevalence estimates for indicators with high measurement error. The improvement in the depression estimate likely reflected correction for underdiagnosis of depression in primary care clinics (25); this underdiagnosis might be because recommendations on comprehensive depression screening in primary care settings took effect only after 2016 in the United States (26). Our calibration model may be helpful for improving estimates for health indicators with similar measurement issues. Similarly, the reduced bias in influenza vaccination estimates likely reflected correction for missing documentation in EHRs of vaccines obtained in nonclinical settings (eg, pharmacies, workplaces) (27,28). Substantial bias (8.4 percentage points) remained in the vaccination indicator even after calibration. Our model might be further improved if the model included additional covariates (eg, employment status, neighborhood pharmacy density) that could better predict vaccination outside of clinics. The degree of improvement resulting from calibration might depend on the magnitude of the misclassification rate (ie, proportion of false-positive and false-negative cases combined). Influenza vaccination had a high misclassification rate (31%), 12 percentage points higher than the misclassification rate for depression, and the calibrated estimate (39.2%) was outside the 95% confidence interval of the 2013–2014 NYC HANES estimate (44.0%–51.3%) (8).

Although some measurement error in EHR data can be eliminated by optimizing the algorithm for defining an indicator (29), some cannot be eliminated (eg, when a condition is underdiagnosed or is not consistently documented in EHRs, when documentation is not up-to-date), and this measurement error requires analytical adjustment. Analytical adjustment may be especially necessary for conditions that are not systematically assessed or recorded across primary care providers in the target population (eg, mental illness). The use of regression-based calibration may be a straightforward approach to addressing this kind of measurement error in EHR data, but it requires collection of and (direct or indirect) linkage to an external gold standard data source and it may be more applicable to aggregate data systems (eg, NYC Macroscope, MDPHnet in Massachusetts [6], the Colorado Health Observation Regional Data Service [30]). In these aggregate data systems, not all types of data are available and thus the options for statistical adjustment are limited. Other statistical approaches may be more appropriate than the statistical approach used in this study if nonaggregate EHR data systems that contain entire individual records are used. Although none of the conditions assessed in this study are overdiagnosed, our calibration model may also be helpful for identifying probable false-positive cases on the basis of patient or provider characteristics and adjusting estimates accordingly. Given that measurement error inherently exists in EHR data for some health indicators, incorporation or automation of calibration or other adjustment procedures into EHR-based surveillance systems may further advance the use of EHRs for actionable public health purposes. These data are useful not only to public health researchers but also to clinicians, as population-level data may inform their decision making on patient care.

This study has several limitations. First, we conducted the model by using a small sample and a small number of covariates. Additional covariates (eg, race/ethnicity [data for which became available only after extraction of 2013 NYC Macroscope data], other clinical conditions) and a larger chart review sample might further enhance calibration performance. Second, we assumed that the conditional probability distribution from the chart review sample could be carried over to the NYC Macroscope sample. Despite similar age and sex distributions, the chart review sample had a larger proportion of persons from the wealthiest neighborhoods than the NYC Macroscope sample had. Our model did not include any adjustment for this difference in sampling distributions between the 2 samples; such an adjustment could further improve the model’s performance. Third, our calibration model is useful only when NYC HANES estimates are close to the true prevalence. The NYC Macroscope may sometimes provide a more reliable estimate. Fourth, the cutoff used in this study for classifying the indicators into high measurement error and low measurement error was arbitrary. Fifth, the 2013–2014 NYC HANES data used for validation (ie, assessing bias) were not independent of the data used for model building and overlapped them. However, in our sensitivity analysis, we found minimal changes in the 2013–2014 NYC HANES estimates after excluding the overlapping data. Despite these limitations, to our knowledge, this is the first study to adopt a calibration approach to address measurement error in EHR-based prevalence estimates from an aggregate EHR data system. Furthermore, the use of a local data source (ie, 2013–2014 NYC HANES) allowed us to obtain local-level regression coefficients.

As EHR data become increasingly available for population health surveillance, it is important to ensure data accuracy; calibration is a potential approach to analytically reducing measurement error in EHR-based prevalence estimates. Appropriate statistical adjustment can expand the utility of EHR data beyond clinical research, widening their applications in public health. Continued effort is warranted for validating and building on the calibration model developed in this study by using other EHR data and additional health indicators.
